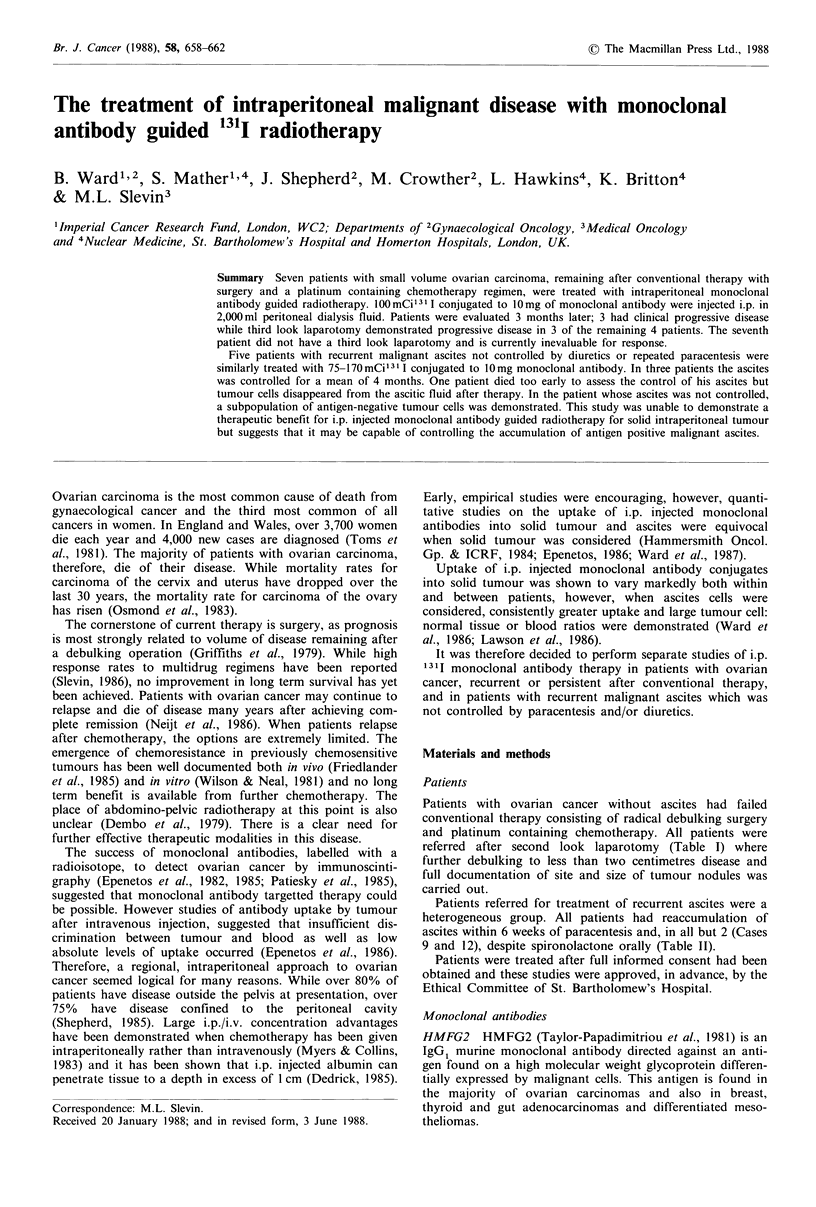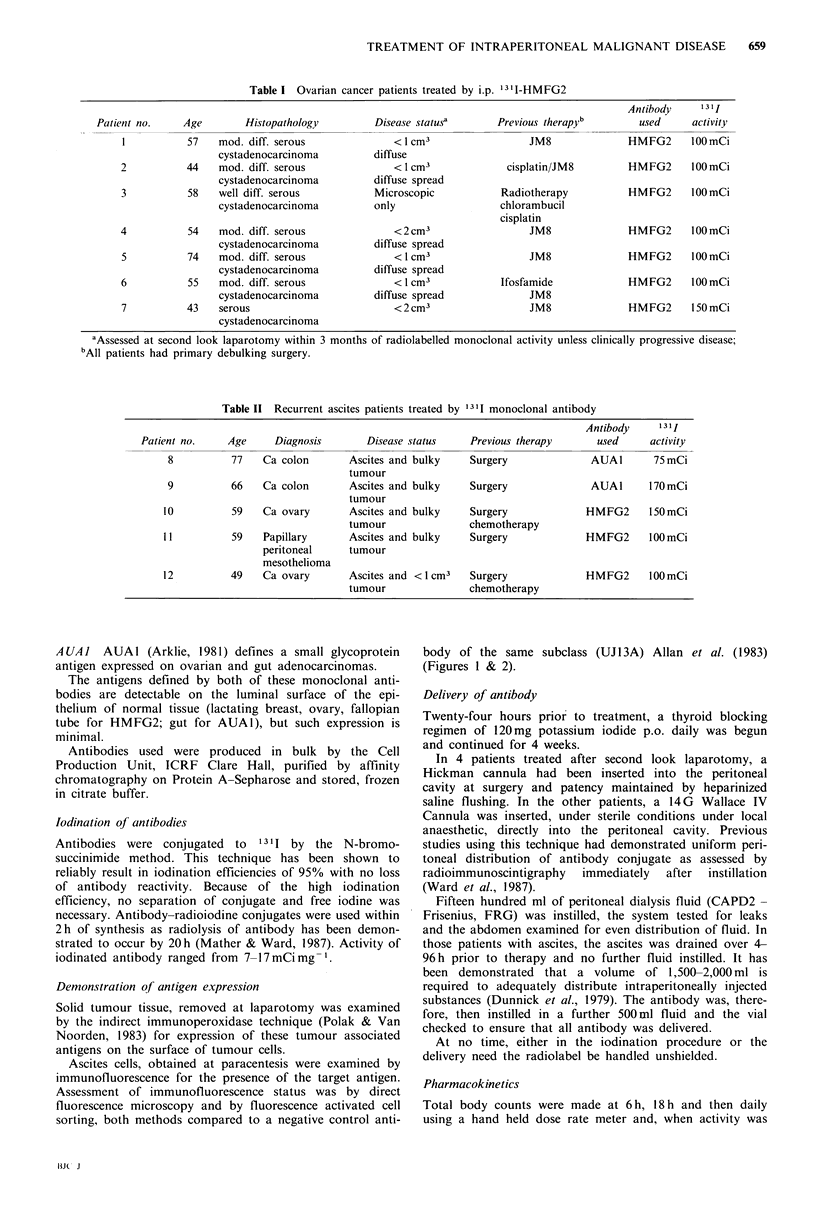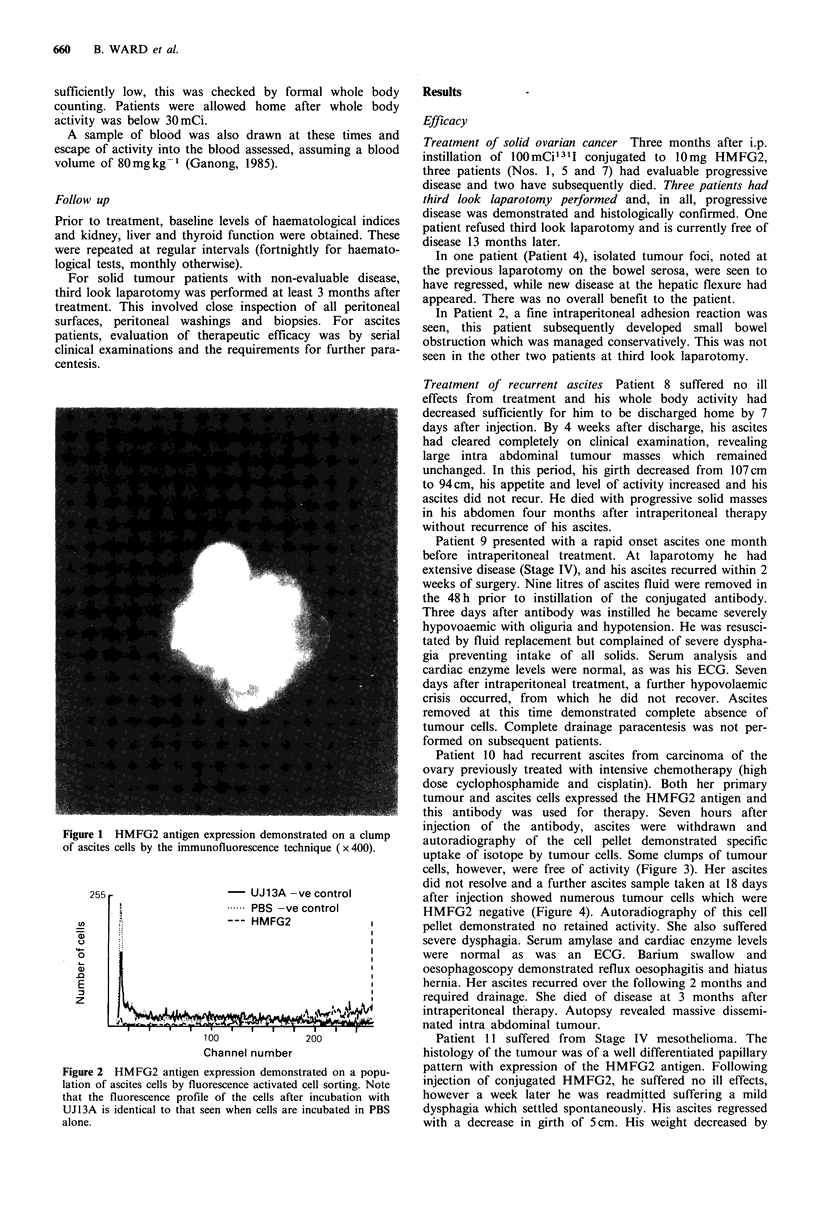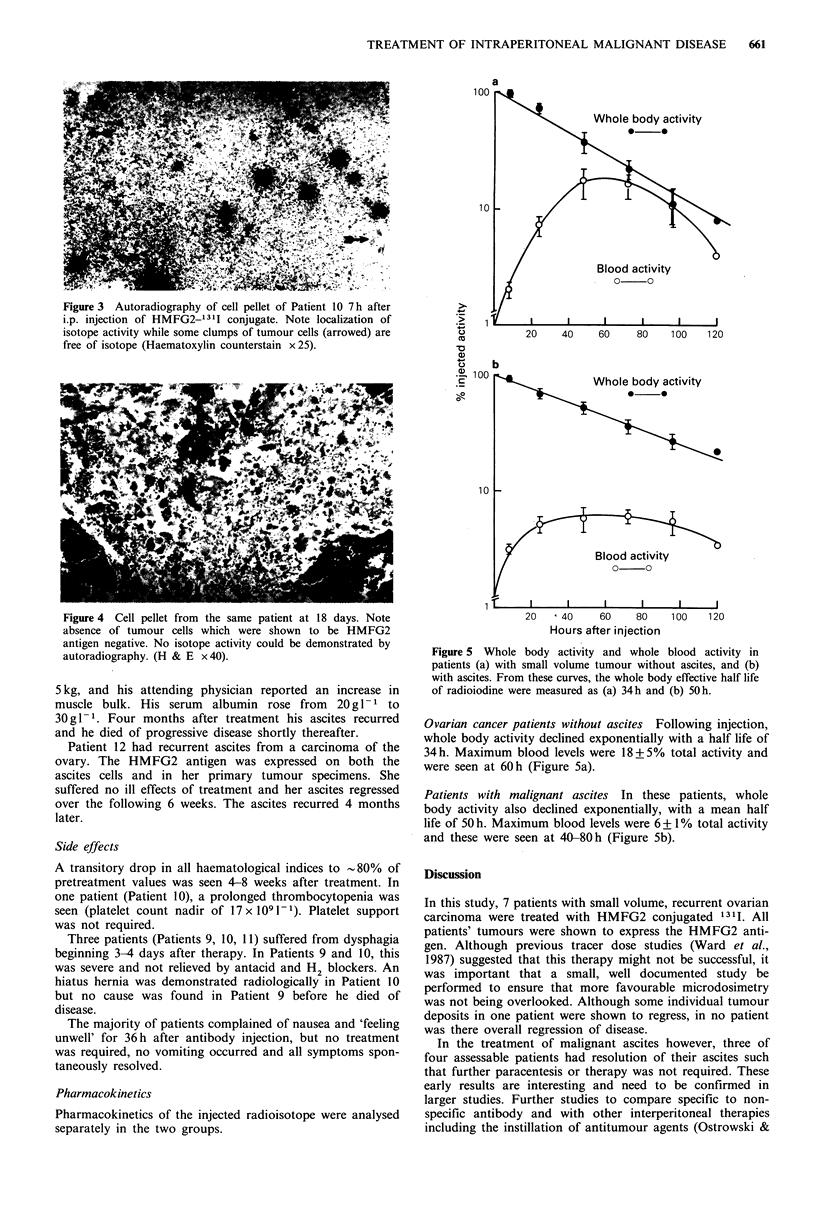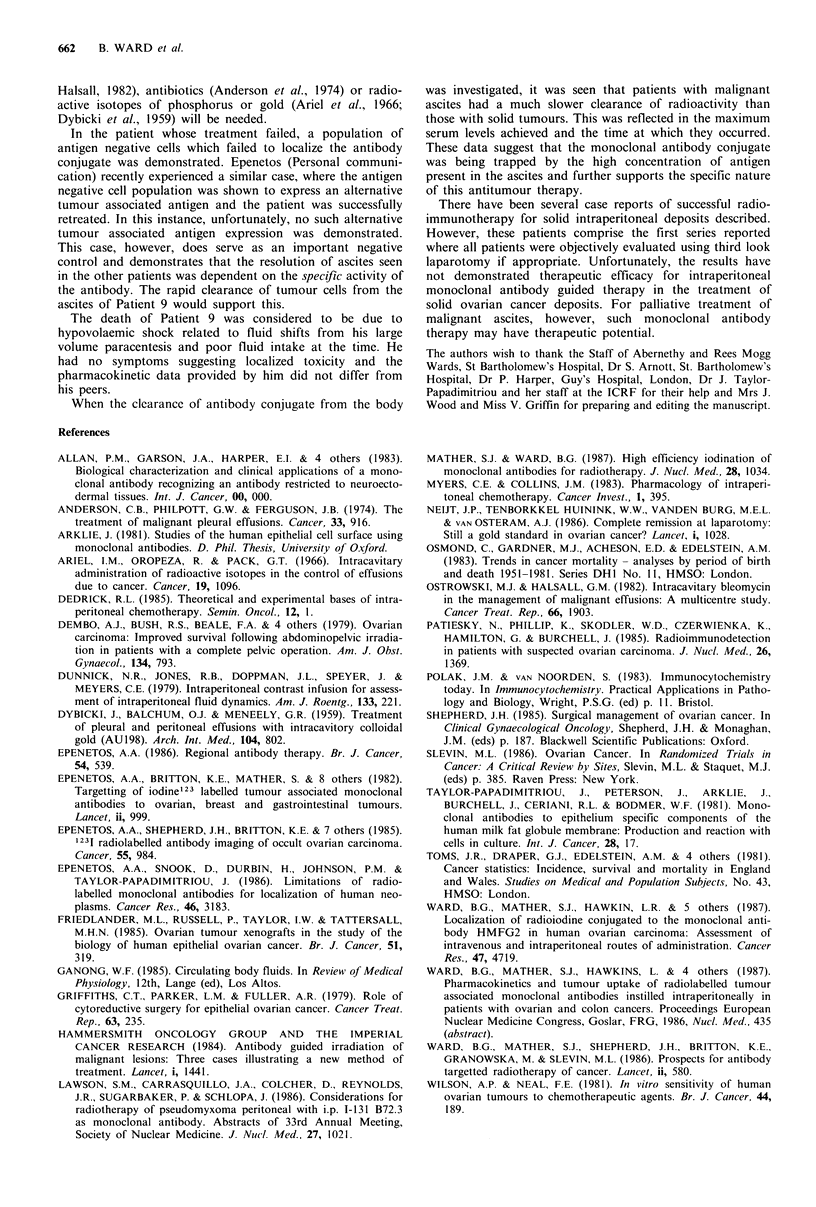# The treatment of intraperitoneal malignant disease with monoclonal antibody guided 131I radiotherapy.

**DOI:** 10.1038/bjc.1988.280

**Published:** 1988-11

**Authors:** B. Ward, S. Mather, J. Shepherd, M. Crowther, L. Hawkins, K. Britton, M. L. Slevin

**Affiliations:** Imperial Cancer Research Fund, London, UK.

## Abstract

**Images:**


*Br. J. Cancer* (1988), **58**, 658–662 © The Macmillan Press Ltd., 1988

### The treatment of intraperitoneal malignant disease with monoclonal antibody guided $^{131}$I radiotherapy

B. Ward[1,2], S. Mather[1,4], J. Shepherd[2], M. Crowther[2], L. Hawkins[4], K. Britton[4] & M.L. Slevin[3]

[1]*Imperial Cancer Research Fund, London, WC2; Departments of* [2]*Gynaecological Oncology,* [3]*Medical Oncology and* [4]*Nuclear Medicine, St. Bartholomew's Hospital and Homerton Hospitals, London, UK.*

**Summary** Seven patients with small volume ovarian carcinoma, remaining after conventional therapy with surgery and a platinum containing chemotherapy regimen, were treated with intraperitoneal monoclonal antibody guided radiotherapy. 100 mCi $^{131}$I conjugated to 10 mg of monoclonal antibody were injected i.p. in 2,000 ml peritoneal dialysis fluid. Patients were evaluated 3 months later; 3 had clinical progressive disease while third look laparotomy demonstrated progressive disease in 3 of the remaining 4 patients. The seventh patient did not have a third look laparotomy and is currently inevaluable for response.

Five patients with recurrent malignant ascites not controlled by diuretics or repeated paracentesis were similarly treated with 75–170 mCi $^{131}$I conjugated to 10 mg monoclonal antibody. In three patients the ascites was controlled for a mean of 4 months. One patient died too early to assess the control of his ascites but tumour cells disappeared from the ascitic fluid after therapy. In the patient whose ascites was not controlled, a subpopulation of antigen-negative tumour cells was demonstrated. This study was unable to demonstrate a therapeutic benefit for i.p. injected monoclonal antibody guided radiotherapy for solid intraperitoneal tumour but suggests that it may be capable of controlling the accumulation of antigen positive malignant ascites.

Ovarian carcinoma is the most common cause of death from gynaecological cancer and the third most common of all cancers in women. In England and Wales, over 3,700 women die each year and 4,000 new cases are diagnosed (Toms *et al.*, 1981). The majority of patients with ovarian carcinoma, therefore, die of their disease. While mortality rates for carcinoma of the cervix and uterus have dropped over the last 30 years, the mortality rate for carcinoma of the ovary has risen (Osmond *et al.*, 1983).

The cornerstone of current therapy is surgery, as prognosis is most strongly related to volume of disease remaining after a debulking operation (Griffiths *et al.*, 1979). While high response rates to multidrug regimens have been reported (Slevin, 1986), no improvement in long term survival has yet been achieved. Patients with ovarian cancer may continue to relapse and die of disease many years after achieving complete remission (Neijt *et al.*, 1986). When patients relapse after chemotherapy, the options are extremely limited. The emergence of chemoresistance in previously chemosensitive tumours has been well documented both *in vivo* (Friedlander *et al.*, 1985) and *in vitro* (Wilson & Neal, 1981) and no long term benefit is available from further chemotherapy. The place of abdomino-pelvic radiotherapy at this point is also unclear (Dembo *et al.*, 1979). There is a clear need for further effective therapeutic modalities in this disease.

The success of monoclonal antibodies, labelled with a radioisotope, to detect ovarian cancer by immunoscintigraphy (Epenetos *et al.*, 1982, 1985; Patiesky *et al.*, 1985), suggested that monoclonal antibody targetted therapy could be possible. However studies of antibody uptake by tumour after intravenous injection, suggested that insufficient discrimination between tumour and blood as well as low absolute levels of uptake occurred (Epenetos *et al.*, 1986). Therefore, a regional, intraperitoneal approach to ovarian cancer seemed logical for many reasons. While over 80% of patients have disease outside the pelvis at presentation, over 75% have disease confined to the peritoneal cavity (Shepherd, 1985). Large i.p./i.v. concentration advantages have been demonstrated when chemotherapy has been given intraperitoneally rather than intravenously (Myers & Collins, 1983) and it has been shown that i.p. injected albumin can penetrate tissue to a depth in excess of 1 cm (Dedrick, 1985).

Correspondence: M.L. Slevin.
Received 20 January 1988; and in revised form, 3 June 1988.

Early, empirical studies were encouraging, however, quantitative studies on the uptake of i.p. injected monoclonal antibodies into solid tumour and ascites were equivocal when solid tumour was considered (Hammersmith Oncol. Gp. & ICRF, 1984; Epenetos, 1986; Ward *et al.*, 1987).

Uptake of i.p. injected monoclonal antibody conjugates into solid tumour was shown to vary markedly both within and between patients, however, when ascites cells were considered, consistently greater uptake and large tumour cell: normal tissue or blood ratios were demonstrated (Ward *et al.*, 1986; Lawson *et al.*, 1986).

It was therefore decided to perform separate studies of i.p. $^{131}$I monoclonal antibody therapy in patients with ovarian cancer, recurrent or persistent after conventional therapy, and in patients with recurrent malignant ascites which was not controlled by paracentesis and/or diuretics.

### Materials and methods

### *Patients*

Patients with ovarian cancer without ascites had failed conventional therapy consisting of radical debulking surgery and platinum containing chemotherapy. All patients were referred after second look laparotomy (Table I) where further debulking to less than two centimetres disease and full documentation of site and size of tumour nodules was carried out.

Patients referred for treatment of recurrent ascites were a heterogeneous group. All patients had reaccumulation of ascites within 6 weeks of paracentesis and, in all but 2 (Cases 9 and 12), despite spironolactone orally (Table II).

Patients were treated after full informed consent had been obtained and these studies were approved, in advance, by the Ethical Committee of St. Bartholomew's Hospital.

### *Monoclonal antibodies*

*HMFG2* HMFG2 (Taylor-Papadimitriou *et al.*, 1981) is an $IgG_1$ murine monoclonal antibody directed against an antigen found on a high molecular weight glycoprotein differentially expressed by malignant cells. This antigen is found in the majority of ovarian carcinomas and also in breast, thyroid and gut adenocarcinomas and differentiated mesotheliomas.

**Table I** Ovarian cancer patients treated by i.p. $^{131}$I-HMFG2

| *Patient no.* | *Age* | *Histopathology* | *Disease status*[a] | *Previous therapy*[b] | *Antibody used* | *$^{131}I$ activity* |
|---|---|---|---|---|---|---|
| 1 | 57 | mod. diff. serous cystadenocarcinoma | $<1\,cm^3$ diffuse | JM8 | HMFG2 | 100 mCi |
| 2 | 44 | mod. diff. serous cystadenocarcinoma | $<1\,cm^3$ diffuse spread | cisplatin/JM8 | HMFG2 | 100 mCi |
| 3 | 58 | well diff. serous cystadenocarcinoma | Microscopic only | Radiotherapy chlorambucil cisplatin | HMFG2 | 100 mCi |
| 4 | 54 | mod. diff. serous cystadenocarcinoma | $<2\,cm^3$ diffuse spread | JM8 | HMFG2 | 100 mCi |
| 5 | 74 | mod. diff. serous cystadenocarcinoma | $<1\,cm^3$ diffuse spread | JM8 | HMFG2 | 100 mCi |
| 6 | 55 | mod. diff. serous cystadenocarcinoma | $<1\,cm^3$ diffuse spread | Ifosfamide JM8 | HMFG2 | 100 mCi |
| 7 | 43 | serous cystadenocarcinoma | $<2\,cm^3$ | JM8 | HMFG2 | 150 mCi |

[a]Assessed at second look laparotomy within 3 months of radiolabelled monoclonal activity unless clinically progressive disease; [b]All patients had primary debulking surgery.

**Table II** Recurrent ascites patients treated by $^{131}$I monoclonal antibody

| *Patient no.* | *Age* | *Diagnosis* | *Disease status* | *Previous therapy* | *Antibody used* | *$^{131}I$ activity* |
|---|---|---|---|---|---|---|
| 8 | 77 | Ca colon | Ascites and bulky tumour | Surgery | AUA1 | 75 mCi |
| 9 | 66 | Ca colon | Ascites and bulky tumour | Surgery | AUA1 | 170 mCi |
| 10 | 59 | Ca ovary | Ascites and bulky tumour | Surgery chemotherapy | HMFG2 | 150 mCi |
| 11 | 59 | Papillary peritoneal mesothelioma | Ascites and bulky tumour | Surgery | HMFG2 | 100 mCi |
| 12 | 49 | Ca ovary | Ascites and $<1\,cm^3$ tumour | Surgery chemotherapy | HMFG2 | 100 mCi |

*AUA1* AUA1 (Arklie, 1981) defines a small glycoprotein antigen expressed on ovarian and gut adenocarcinomas.

The antigens defined by both of these monoclonal antibodies are detectable on the luminal surface of the epithelium of normal tissue (lactating breast, ovary, fallopian tube for HMFG2; gut for AUA1), but such expression is minimal.

Antibodies used were produced in bulk by the Cell Production Unit, ICRF Clare Hall, purified by affinity chromatography on Protein A–Sepharose and stored, frozen in citrate buffer.

*Iodination of antibodies*

Antibodies were conjugated to $^{131}$I by the N-bromosuccinimide method. This technique has been shown to reliably result in iodination efficiencies of 95% with no loss of antibody reactivity. Because of the high iodination efficiency, no separation of conjugate and free iodine was necessary. Antibody–radioiodine conjugates were used within 2 h of synthesis as radiolysis of antibody has been demonstrated to occur by 20 h (Mather & Ward, 1987). Activity of iodinated antibody ranged from 7–17 $mCi\,mg^{-1}$.

*Demonstration of antigen expression*

Solid tumour tissue, removed at laparotomy was examined by the indirect immunoperoxidase technique (Polak & Van Noorden, 1983) for expression of these tumour associated antigens on the surface of tumour cells.

Ascites cells, obtained at paracentesis were examined by immunofluorescence for the presence of the target antigen. Assessment of immunofluorescence status was by direct fluorescence microscopy and by fluorescence activated cell sorting, both methods compared to a negative control antibody of the same subclass (UJ13A) Allan *et al.* (1983) (Figures 1 & 2).

*Delivery of antibody*

Twenty-four hours prior to treatment, a thyroid blocking regimen of 120 mg potassium iodide p.o. daily was begun and continued for 4 weeks.

In 4 patients treated after second look laparotomy, a Hickman cannula had been inserted into the peritoneal cavity at surgery and patency maintained by heparinized saline flushing. In the other patients, a 14 G Wallace IV Cannula was inserted, under sterile conditions under local anaesthetic, directly into the peritoneal cavity. Previous studies using this technique had demonstrated uniform peritoneal distribution of antibody conjugate as assessed by radioimmunoscintigraphy immediately after instillation (Ward *et al.*, 1987).

Fifteen hundred ml of peritoneal dialysis fluid (CAPD2 – Frisenius, FRG) was instilled, the system tested for leaks and the abdomen examined for even distribution of fluid. In those patients with ascites, the ascites was drained over 4–96 h prior to therapy and no further fluid instilled. It has been demonstrated that a volume of 1,500–2,000 ml is required to adequately distribute intraperitoneally injected substances (Dunnick *et al.*, 1979). The antibody was, therefore, then instilled in a further 500 ml fluid and the vial checked to ensure that all antibody was delivered.

At no time, either in the iodination procedure or the delivery need the radiolabel be handled unshielded.

*Pharmacokinetics*

Total body counts were made at 6 h, 18 h and then daily using a hand held dose rate meter and, when activity was sufficiently low, this was checked by formal whole body counting. Patients were allowed home after whole body activity was below 30 mCi.

A sample of blood was also drawn at these times and escape of activity into the blood assessed, assuming a blood volume of 80 mg kg$^{-1}$ (Ganong, 1985).

*Follow up*

Prior to treatment, baseline levels of haematological indices and kidney, liver and thyroid function were obtained. These were repeated at regular intervals (fortnightly for haematological tests, monthly otherwise).

For solid tumour patients with non-evaluable disease, third look laparotomy was performed at least 3 months after treatment. This involved close inspection of all peritoneal surfaces, peritoneal washings and biopsies. For ascites patients, evaluation of therapeutic efficacy was by serial clinical examinations and the requirements for further paracentesis.

**Figure 1** HMFG2 antigen expression demonstrated on a clump of ascites cells by the immunofluorescence technique (×400).

**Figure 2** HMFG2 antigen expression demonstrated on a population of ascites cells by fluorescence activated cell sorting. Note that the fluorescence profile of the cells after incubation with UJ13A is identical to that seen when cells are incubated in PBS alone.

### Results

*Efficacy*

*Treatment of solid ovarian cancer* Three months after i.p. instillation of 100 mCi$^{131}$I conjugated to 10 mg HMFG2, three patients (Nos. 1, 5 and 7) had evaluable progressive disease and two have subsequently died. *Three patients had third look laparotomy performed* and, in all, progressive disease was demonstrated and histologically confirmed. One patient refused third look laparotomy and is currently free of disease 13 months later.

In one patient (Patient 4), isolated tumour foci, noted at the previous laparotomy on the bowel serosa, were seen to have regressed, while new disease at the hepatic flexure had appeared. There was no overall benefit to the patient.

In Patient 2, a fine intraperitoneal adhesion reaction was seen, this patient subsequently developed small bowel obstruction which was managed conservatively. This was not seen in the other two patients at third look laparotomy.

*Treatment of recurrent ascites* Patient 8 suffered no ill effects from treatment and his whole body activity had decreased sufficiently for him to be discharged home by 7 days after injection. By 4 weeks after discharge, his ascites had cleared completely on clinical examination, revealing large intra abdominal tumour masses which remained unchanged. In this period, his girth decreased from 107 cm to 94 cm, his appetite and level of activity increased and his ascites did not recur. He died with progressive solid masses in his abdomen four months after intraperitoneal therapy without recurrence of his ascites.

Patient 9 presented with a rapid onset ascites one month before intraperitoneal treatment. At laparotomy he had extensive disease (Stage IV), and his ascites recurred within 2 weeks of surgery. Nine litres of ascites fluid were removed in the 48 h prior to instillation of the conjugated antibody. Three days after antibody was instilled he became severely hypovoaemic with oliguria and hypotension. He was resuscitated by fluid replacement but complained of severe dysphagia preventing intake of all solids. Serum analysis and cardiac enzyme levels were normal, as was his ECG. Seven days after intraperitoneal treatment, a further hypovolaemic crisis occurred, from which he did not recover. Ascites removed at this time demonstrated complete absence of tumour cells. Complete drainage paracentesis was not performed on subsequent patients.

Patient 10 had recurrent ascites from carcinoma of the ovary previously treated with intensive chemotherapy (high dose cyclophosphamide and cisplatin). Both her primary tumour and ascites cells expressed the HMFG2 antigen and this antibody was used for therapy. Seven hours after injection of the antibody, ascites were withdrawn and autoradiography of the cell pellet demonstrated specific uptake of isotope by tumour cells. Some clumps of tumour cells, however, were free of activity (Figure 3). Her ascites did not resolve and a further ascites sample taken at 18 days after injection showed numerous tumour cells which were HMFG2 negative (Figure 4). Autoradiography of this cell pellet demonstrated no retained activity. She also suffered severe dysphagia. Serum amylase and cardiac enzyme levels were normal as was an ECG. Barium swallow and oesophagoscopy demonstrated reflux oesophagitis and hiatus hernia. Her ascites recurred over the following 2 months and required drainage. She died of disease at 3 months after intraperitoneal therapy. Autopsy revealed massive disseminated intra abdominal tumour.

Patient 11 suffered from Stage IV mesothelioma. The histology of the tumour was of a well differentiated papillary pattern with expression of the HMFG2 antigen. Following injection of conjugated HMFG2, he suffered no ill effects, however a week later he was readmitted suffering a mild dysphagia which settled spontaneously. His ascites regressed with a decrease in girth of 5 cm. His weight decreased by

**Figure 3** Autoradiography of cell pellet of Patient 10 7 h after i.p. injection of HMFG2–$^{131}$I conjugate. Note localization of isotope activity while some clumps of tumour cells (arrowed) are free of isotope (Haematoxylin counterstain ×25).

**Figure 4** Cell pellet from the same patient at 18 days. Note absence of tumour cells which were shown to be HMFG2 antigen negative. No isotope activity could be demonstrated by autoradiography. (H & E ×40).

5 kg, and his attending physician reported an increase in muscle bulk. His serum albumin rose from 20 g $l^{-1}$ to 30 g $l^{-1}$. Four months after treatment his ascites recurred and he died of progressive disease shortly thereafter.

Patient 12 had recurrent ascites from a carcinoma of the ovary. The HMFG2 antigen was expressed on both the ascites cells and in her primary tumour specimens. She suffered no ill effects of treatment and her ascites regressed over the following 6 weeks. The ascites recurred 4 months later.

*Side effects*

A transitory drop in all haematological indices to ~80% of pretreatment values was seen 4–8 weeks after treatment. In one patient (Patient 10), a prolonged thrombocytopenia was seen (platelet count nadir of $17 \times 10^9 l^{-1}$). Platelet support was not required.

Three patients (Patients 9, 10, 11) suffered from dysphagia beginning 3–4 days after therapy. In Patients 9 and 10, this was severe and not relieved by antacid and $H_2$ blockers. An hiatus hernia was demonstrated radiologically in Patient 10 but no cause was found in Patient 9 before he died of disease.

The majority of patients complained of nausea and 'feeling unwell' for 36 h after antibody injection, but no treatment was required, no vomiting occurred and all symptoms spontaneously resolved.

*Pharmacokinetics*

Pharmacokinetics of the injected radioisotope were analysed separately in the two groups.

**Figure 5** Whole body activity and whole blood activity in patients (a) with small volume tumour without ascites, and (b) with ascites. From these curves, the whole body effective half life of radioiodine were measured as (a) 34 h and (b) 50 h.

*Ovarian cancer patients without ascites* Following injection, whole body activity declined exponentially with a half life of 34 h. Maximum blood levels were 18±5% total activity and were seen at 60 h (Figure 5a).

*Patients with malignant ascites* In these patients, whole body activity also declined exponentially, with a mean half life of 50 h. Maximum blood levels were 6±1% total activity and these were seen at 40–80 h (Figure 5b).

### Discussion

In this study, 7 patients with small volume, recurrent ovarian carcinoma were treated with HMFG2 conjugated $^{131}$I. All patients' tumours were shown to express the HMFG2 antigen. Although previous tracer dose studies (Ward *et al.*, 1987) suggested that this therapy might not be successful, it was important that a small, well documented study be performed to ensure that more favourable microdosimetry was not being overlooked. Although some individual tumour deposits in one patient were shown to regress, in no patient was there overall regression of disease.

In the treatment of malignant ascites however, three of four assessable patients had resolution of their ascites such that further paracentesis or therapy was not required. These early results are interesting and need to be confirmed in larger studies. Further studies to compare specific to non-specific antibody and with other interperitoneal therapies including the instillation of antitumour agents (Ostrowski &

Halsall, 1982), antibiotics (Anderson *et al.*, 1974) or radioactive isotopes of phosphorus or gold (Ariel *et al.*, 1966; Dybicki *et al.*, 1959) will be needed.

In the patient whose treatment failed, a population of antigen negative cells which failed to localize the antibody conjugate was demonstrated. Epenetos (Personal communication) recently experienced a similar case, where the antigen negative cell population was shown to express an alternative tumour associated antigen and the patient was successfully retreated. In this instance, unfortunately, no such alternative tumour associated antigen expression was demonstrated. This case, however, does serve as an important negative control and demonstrates that the resolution of ascites seen in the other patients was dependent on the *specific* activity of the antibody. The rapid clearance of tumour cells from the ascites of Patient 9 would support this.

The death of Patient 9 was considered to be due to hypovolaemic shock related to fluid shifts from his large volume paracentesis and poor fluid intake at the time. He had no symptoms suggesting localized toxicity and the pharmacokinetic data provided by him did not differ from his peers.

When the clearance of antibody conjugate from the body was investigated, it was seen that patients with malignant ascites had a much slower clearance of radioactivity than those with solid tumours. This was reflected in the maximum serum levels achieved and the time at which they occurred. These data suggest that the monoclonal antibody conjugate was being trapped by the high concentration of antigen present in the ascites and further supports the specific nature of this antitumour therapy.

There have been several case reports of successful radioimmunotherapy for solid intraperitoneal deposits described. However, these patients comprise the first series reported where all patients were objectively evaluated using third look laparotomy if appropriate. Unfortunately, the results have not demonstrated therapeutic efficacy for intraperitoneal monoclonal antibody guided therapy in the treatment of solid ovarian cancer deposits. For palliative treatment of malignant ascites, however, such monoclonal antibody therapy may have therapeutic potential.

The authors wish to thank the Staff of Abernethy and Rees Mogg Wards, St Bartholomew's Hospital, Dr S. Arnott, St. Bartholomew's Hospital, Dr P. Harper, Guy's Hospital, London, Dr J. Taylor-Papadimitriou and her staff at the ICRF for their help and Mrs J. Wood and Miss V. Griffin for preparing and editing the manuscript.

### References

ALLAN, P.M., GARSON, J.A., HARPER, E.I. & 4 others (1983). Biological characterization and clinical applications of a monoclonal antibody recognizing an antibody restricted to neuroectodermal tissues. *Int. J. Cancer*, **00**, 000.

ANDERSON, C.B., PHILPOTT, G.W. & FERGUSON, J.B. (1974). The treatment of malignant pleural effusions. *Cancer*, **33**, 916.

ARKLIE, J. (1981). Studies of the human epithelial cell surface using monoclonal antibodies. *D. Phil. Thesis, University of Oxford.*

ARIEL, I.M., OROPEZA, R. & PACK, G.T. (1966). Intracavitary administration of radioactive isotopes in the control of effusions due to cancer. *Cancer*, **19**, 1096.

DEDRICK, R.L. (1985). Theoretical and experimental bases of intraperitoneal chemotherapy. *Semin. Oncol.*, **12**, 1.

DEMBO, A.J., BUSH, R.S., BEALE, F.A. & 4 others (1979). Ovarian carcinoma: Improved survival following abdominopelvic irradiation in patients with a complete pelvic operation. *Am. J. Obst. Gynaecol.*, **134**, 793.

DUNNICK, N.R., JONES, R.B., DOPPMAN, J.L., SPEYER, J. & MEYERS, C.E. (1979). Intraperitoneal contrast infusion for assessment of intraperitoneal fluid dynamics. *Am. J. Roentg.*, **133**, 221.

DYBICKI, J., BALCHUM, O.J. & MENEELY, G.R. (1959). Treatment of pleural and peritoneal effusions with intracavitory colloidal gold (AU198). *Arch. Int. Med.*, **104**, 802.

EPENETOS, A.A. (1986). Regional antibody therapy. *Br. J. Cancer*, **54**, 539.

EPENETOS, A.A., BRITTON, K.E., MATHER, S. & 8 others (1982). Targetting of iodine$^{123}$ labelled tumour associated monoclonal antibodies to ovarian, breast and gastrointestinal tumours. *Lancet*, **ii**, 999.

EPENETOS, A.A., SHEPHERD, J.H., BRITTON, K.E. & 7 others (1985). $^{123}$I radiolabelled antibody imaging of occult ovarian carcinoma. *Cancer*, **55**, 984.

EPENETOS, A.A., SNOOK, D., DURBIN, H., JOHNSON, P.M. & TAYLOR-PAPADIMITRIOU, J. (1986). Limitations of radiolabelled monoclonal antibodies for localization of human neoplasms. *Cancer Res.*, **46**, 3183.

FRIEDLANDER, M.L., RUSSELL, P., TAYLOR, I.W. & TATTERSALL, M.H.N. (1985). Ovarian tumour xenografts in the study of the biology of human epithelial ovarian cancer. *Br. J. Cancer*, **51**, 319.

GANONG, W.F. (1985). Circulating body fluids. In *Review of Medical Physiology*, 12th, Lange (ed), Los Altos.

GRIFFITHS, C.T., PARKER, L.M. & FULLER, A.R. (1979). Role of cytoreductive surgery for epithelial ovarian cancer. *Cancer Treat. Rep.*, **63**, 235.

HAMMERSMITH ONCOLOGY GROUP AND THE IMPERIAL CANCER RESEARCH (1984). Antibody guided irradiation of malignant lesions: Three cases illustrating a new method of treatment. *Lancet*, **i**, 1441.

LAWSON, S.M., CARRASQUILLO, J.A., COLCHER, D., REYNOLDS, J.R., SUGARBAKER, P. & SCHLOPA, J. (1986). Considerations for radiotherapy of pseudomyxoma peritoneal with i.p. I-131 B72.3 as monoclonal antibody. Abstracts of 33rd Annual Meeting, Society of Nuclear Medicine. *J. Nucl. Med.*, **27**, 1021.

MATHER, S.J. & WARD, B.G. (1987). High efficiency iodination of monoclonal antibodies for radiotherapy. *J. Nucl. Med.*, **28**, 1034.

MYERS, C.E. & COLLINS, J.M. (1983). Pharmacology of intraperitoneal chemotherapy. *Cancer Invest.*, **1**, 395.

NEIJT, J.P., TENBORKKEL HUININK, W.W., VANDEN BURG, M.E.L. & VAN OSTERAM, A.J. (1986). Complete remission at laparotomy: Still a gold standard in ovarian cancer? *Lancet*, **i**, 1028.

OSMOND, C., GARDNER, M.J., ACHESON, E.D. & EDELSTEIN, A.M. (1983). Trends in cancer mortality – analyses by period of birth and death 1951–1981. Series DH1 No. 11, HMSO: London.

OSTROWSKI, M.J. & HALSALL, G.M. (1982). Intracavitary bleomycin in the management of malignant effusions: A multicentre study. *Cancer Treat. Rep.*, **66**, 1903.

PATIESKY, N., PHILLIP, K., SKODLER, W.D., CZERWIENKA, K., HAMILTON, G. & BURCHELL, J. (1985). Radioimmunodetection in patients with suspected ovarian carcinoma. *J. Nucl. Med.*, **26**, 1369.

POLAK, J.M. & VAN NOORDEN, S. (1983). Immunocytochemistry today. In *Immunocytochemistry*. Practical Applications in Pathology and Biology, Wright, P.S.G. (ed) p. 11. Bristol.

SHEPHERD, J.H. (1985). Surgical management of ovarian cancer. In *Clinical Gynaecological Oncology*, Shepherd, J.H. & Monaghan, J.M. (eds) p. 187. Blackwell Scientific Publications: Oxford.

SLEVIN, M.L. (1986). Ovarian Cancer. In *Randomized Trials in Cancer: A Critical Review by Sites*, Slevin, M.L. & Staquet, M.J. (eds) p. 385. Raven Press: New York.

TAYLOR-PAPADIMITRIOU, J., PETERSON, J., ARKLIE, J., BURCHELL, J., CERIANI, R.L. & BODMER, W.F. (1981). Monoclonal antibodies to epithelium specific components of the human milk fat globule membrane: Production and reaction with cells in culture. *Int. J. Cancer*, **28**, 17.

TOMS, J.R., DRAPER, G.J., EDELSTEIN, A.M. & 4 others (1981). Cancer statistics: Incidence, survival and mortality in England and Wales. *Studies on Medical and Population Subjects*, No. 43, HMSO: London.

WARD, B.G., MATHER, S.J., HAWKIN, L.R. & 5 others (1987). Localization of radioiodine conjugated to the monoclonal antibody HMFG2 in human ovarian carcinoma: Assessment of intravenous and intraperitoneal routes of administration. *Cancer Res.*, **47**, 4719.

WARD, B.G., MATHER, S.J., HAWKINS, L. & 4 others (1987). Pharmacokinetics and tumour uptake of radiolabelled tumour associated monoclonal antibodies instilled intraperitoneally in patients with ovarian and colon cancers. Proceedings European Nuclear Medicine Congress, Goslar, FRG, 1986, *Nucl. Med.*, 435 (*abstract*).

WARD, B.G., MATHER, S.J., SHEPHERD, J.H., BRITTON, K.E., GRANOWSKA, M. & SLEVIN, M.L. (1986). Prospects for antibody targetted radiotherapy of cancer. *Lancet*, **ii**, 580.

WILSON, A.P. & NEAL, F.E. (1981). *In vitro* sensitivity of human ovarian tumours to chemotherapeutic agents. *Br. J. Cancer*, **44**, 189.